# Ionic liquid/TiO_2_ nanoparticles doped with non-expensive metals: new active catalyst for phenol photodegradation

**DOI:** 10.1039/d1ra08459c

**Published:** 2022-01-18

**Authors:** Daiane Kessler Fischer, Karina Rodrigues de Fraga, Carla Weber Scheeren

**Affiliations:** Laboratory of Catalysis and Nanomaterials, School of Chemistry and Food, Federal University of Rio Grande-FURG Rua Barão do Caí, 125 CEP 95500-000 Santo Antônio da Patrulha RS Brazil carlascheeren@gmail.com

## Abstract

TiO_2_ nanoparticles were synthesized using 1-*n*-butyl-3-methylimidazolium tetrafluoroborate (BMI·BF_4_) ionic liquid and doped with non-expensive metals Cu^2+^ and Fe^3+^ by the sol–gel method. The new generated photocatalysts had their morphological, textural and structural characteristics analysed by scanning electron microscopy and dispersive X-ray spectroscopy (SEM/EDS), transmission electron microscopy (TEM), Brunauer–Emmett–Teller analysis (BET), Fourier transform infrared spectroscopy (FTIR), X-ray diffraction (XRD) and diffuse reflectance spectroscopy (DRS). The results showed two phases by XRD analysis, anatase (majority) and rutile (minority). The SEM micrographs exposed spherical TiO_2_ NPs/BMI·BF_4_ IL and compact layers for Cu^2+^ and Fe^3+^-doped TiO_2_ NPs in BMI·BF_4_ IL, the EDX confirmed only the presence of Ti, O, Fe and Cu. The BET and BJH analyses exhibited high porous TiO_2_ NPs/BMI·BF_4_ IL. The BET and BJH analyses confirmed that the pore diameter of mesoporous materials was between 12 and 16 nm with similar values for surface area (55–63 m^2^ g^−1^). The TEM images exhibited spherical shape nanoparticles with mean diameter of 20–22 nm. The DRS analysis and Tauc equation were applied to estimate the optical energy band gap of the photocatalysts. The energy band gap values of 3.1 eV, 3.32 eV, and 2.78 eV were obtained for TiO_2_ NPs/BMI·BF_4_ IL, 1% Fe^3+^-doped TiO_2_ NPs/BMI·BF_4_ IL and 1% Cu^2+^-doped TiO_2_ NPs/BMI·BF_4_ IL, respectively. Phenol photodegradation was realized using Cu^2+^ and Fe^3+^-doped TiO_2_ NPs/BMI·BF_4_ IL under UV/visible irradiation and quantified by HPLC-FLD. The phenol photodegradation was investigated by different concentrations of metal-doped TiO_2_ NPs/BMI·BF_4_ IL. The new active photocatalysts 1% Cu^2+^-doped TiO_2_ NPs and 1% Fe^3+^-doped TiO_2_ NPs/BMI·BF_4_ IL exhibited high catalytic activity (99.9% and 96.8%, respectively). The photocatalysts 1% Cu^2+^ and 1% Fe^3+^-doped TiO_2_ NPs/BMI·BF_4_ IL were also evaluated using industrial wastewater from the tobacco industry. The results showed 56.7% phenol photodegradation, due to the complexity of the tobacco matrix wastewater.

## Abstract 2

TiO_2_ nanoparticles were synthesized using 1-*n*-butyl-3-methylimidazolium tetrafluoroborate (BMI·BF_4_) ionic liquid and doped with non-expensive metals Cu^2+^ and Fe^3+^ by the sol–gel method.

## Graphical abstract

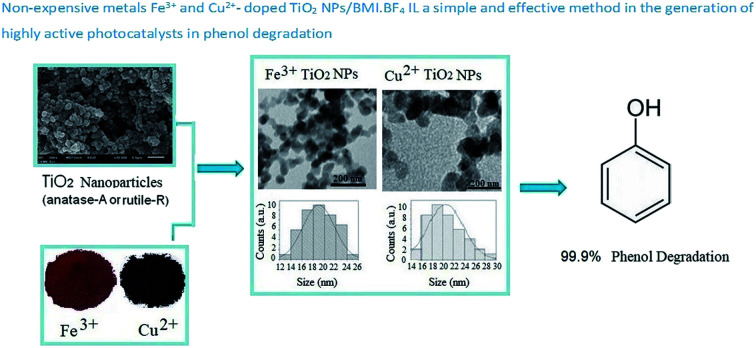

## 1. Introduction

Phenolic compounds represent an important class of polluting organic molecules present in wastewater.^1^ The pesticide, chemical, petrochemical, paint, textile, and biotechnological industries and food processing, generate these compounds.^2^ These compounds are highly toxic and their presence prevents the activity of microorganisms in biological wastewater, reducing the biodegradation of other components.^3,4^ Several technologies exhibit phenol degradation in wastewater.^5^ As an example, we can cite biological treatment, activated carbon adsorption and advanced oxidative processes (AOPs).^6–12^ Photocatalysis stands out among the advanced oxidative process (AOP) techniques. This consists of the activation of a semiconductor by sunlight or artificial light. One of the main semiconductors used is TiO_2_, which demonstrates efficiency in the photodegradation of organic compounds.^11,13,14^

TiO_2_ has been shown to be one of the most suitable catalysts for environmental applications, considering its biological and chemical inertness, strong oxidizing power, non-toxicity, insolubility and stability against chemical corrosion.^9–11^ This semiconductor when at the nanoscale has a pronounced effect on its photocatalytic properties, due to its larger surface area, exhibiting a high percentage of its constitutive atoms on the particle surface.^13^ The metal ion addition to TiO_2_ provides good control of the main particle size to produce nanocatalysts.^14^ The doping of TiO_2_ by transition metal cations is an efficient strategy to reduce the electron–hole pair recombination rate and increase photocatalytic efficiency.^9^

Supported ionic liquid phase (SILP) technology is emerging as an interesting protocol for the immobilization of metal catalysts because it may combine the advantages of ionic liquids (IL) with those of heterogeneous support materials. These materials are prepared by the covalent attachment of IL to the support surface or by the deposition of phases containing catalytically active species—usually transition metal compounds on the surface of the support, which is usually a silica, alumina polymeric material and titanium dioxide.^15^ Ionic liquids (ILs), containing large anions and organic cations, have attracted special attention due to their unique properties, such as good dissolving ability, low volatility, high chemical and thermal stability, ionic conductivity and wide electrochemical window.^16^ ILs have been widely used as templates, solvents, or reactants for the functionalization of nanomaterials with improving the catalytic activity.^17,18^ For example, researchers have reported an efficient microwave-assisted ionothermal recipe for the preparation of anatase TiO_2_ single crystal photocatalyst with tunable percentage of reactive facets.^19^ In another investigation, a simple method was proposed for the fabrication of high quality TiO_2_ nanocrystals in IL.^20^ A study about the effect of IL as an important role in accelerating electron transfer, when it is adsorbed at the electrochemical interface, exhibited to be beneficial for tuning the electrocatalytic properties of carbon nanotubes (CNTs)/IL/Pt hybrids.^17^

Noble metals like Au are the most studied; but other metals like palladium, copper and iron has shown to be a useful for photocatalytic reactions. The sol–gel method is widely used to prepare metal ion doped TiO_2_ due to its ease of controlling pore structures and concentration.^21^ When TiO_2_ undergoes UV-vis irradiation with energy equal to or greater than the energy of the semiconductor band gap, the valence band electrons are excited to the conduction band, generating an electron–hole pair. The hole formed has an extremely oxidizing potential. The water adsorbed on the TiO_2_ surface interacts with the electron–hole pairs generating the free radical entities (hydroxyl, peroxide, mainly), which reduce the organic matter.^22–25^

In photocatalytic studies, the optical features of the material exhibit an important function to turn light into chemical reactions. The optical response is estimated by the calculation of its band gap, which represents the energy required to move an electron in a bound state in the valence band to the conduction band, generating electrically bounded electron–hole pairs.^26^

In this work, we conducted the study of the photocatalytic properties of TiO_2_ nanoparticles in BMI·BF_4_ ionic liquid doped with non-expensive metals Cu^2+^ and Fe^3+^. The new photocatalysts formed were applied in phenol photodegradation standard and industrial wastewater samples, thus evaluating the doping effect and the photocatalytic activity. The photocatalysts showed higher photocatalytic activity in both samples and were characterized by scanning electron microscopy (SEM), dispersive energy spectroscopy (EDS), transmission electron microscopy (TEM), surface area analysis (BET), Fourier transform infrared spectroscopy (FTIR) and X-ray diffraction (XRD) to determine their structural and morphological structure.

## 2. Experimental

### 2.1. Synthesis of Fe^3+^ and Cu^2+^-doped TiO_2_ NPs in BMI·BF_4_ IL

**Scheme 1:**
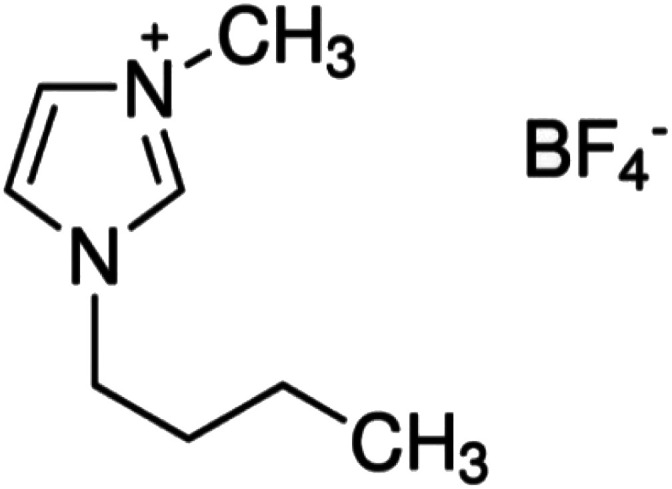
Molecular structure of 1-*n*-butyl-3-methylimidazolium tetrafluoroborate ionic liquid (BMI·BF_4_).

The Fe^3+^ and Cu^2+^-doped TiO_2_ NPs in BMI·BF_4_ IL occurred by mixing of ethanol (25 mL) and titanium tetraisopropoxide (TTIP) (2.5 mL) under stirring during 10 minutes in a bottle with addition of 1 mL of in BMI·BF_4_ IL.^27^ For pH adjusting, sulfuric acid solution (0.125 mL) was added dropwise to the solution, and stirring was continued for 30 minutes. Then, 25 mL double distilled water and 121 mL absolute propanol were stirred and added dropwise to the solution. For doped TiO_2_ NPs in BMI·BF_4_ IL, Fe(SO_4_)_3_·5H_2_O or Cu(SO_4_)·5H_2_O were added to this solution in different atomic ratios (Table 1) and the stirring continued for 90 minutes. For the gel formation and exit of the alcohol, the formed sol was stirred at room temperature for 24 h; after that, the gel was dried under vacuum at 90 °C for about 12 h and then calcined at 600 °C for 1 h.^27^ The molecular structure of 1-*n*-butyl-3-methylimidazolium tetrafluoroborate (BMI·BF_4_) IL is exposed in Scheme 1.

**Table 1:** Metal precursor and metal-doped TiO_2_ NPs/BMI·BF_4_ IL percentage

Metal precursor	Metal (m/m)
—	NPsTiO_2_
CuSO_4_·5H_2_O	0.5% Cu^2+^
CuSO_4_·5H_2_O	1% Cu^2+^
CuSO_4_·5H_2_O	5% Cu^2+^
Fe_2_(SO_4_)_3_·5H_2_O	0.5% Fe^3+^
Fe_2_(SO_4_)_3_·5H_2_O	1% Fe^3+^
Fe_2_(SO_4_)_3_·5H_2_O	5% Fe^3+^
Fe_2_(SO_4_)_3_·5H_2_O	10% Fe^3+^

The metal precursors concentrations used in the synthesis of catalysts are shown in Table 1.

### 2.2. Phenol photodegradation

Phenol photodegradation was performed using a 250 mL photo reactor composed of a stirring system, digital thermometer and a UV light source (consisting of a low-pressure mercury vapor lamp of 150 W, from which the bulb was removed). In the experimental photocatalytic degradation of aforementioned concentrations of phenol (10 mg L^−1^) at pH 3 and using different atomic ratios (0.5; 1; 5 and 10%), metal-doped TiO_2_ NPs under UV irradiation (380 nm) was investigated. The irradiation time was 180 minutes and aliquots of solution were collected from the reactor at 15 minutes intervals. In the experiments, the magnetic stirrer was used to maintain the uniformity of suspensions. The samples were centrifuged (6000 rpm for 5 min) and filtered to separate Fe^3+^ or Cu^2+^-doped TiO_2_ particles. The aliquots were analyzed by HPLC-FLD (Agilent brand, model 1260). The injections of the samples were performed according to the established parameters, with a flow of 1.5 mL min^−1^, running time of 35 minutes, post-run of 10 minutes, injection volume 20 μL, LiChrospher RP column 18 and 5 μm, 250 × 4 mm and guard column C18, 4 × 3 mm. The results were quantified based on the peak area, using ChemStation software.^28^

## 3. Characterization

### 3.1. Fourier transform infrared spectrophotometer (FTIR)

The samples were analyzed in the infrared region with readings from 4000 to 400 cm^−1^ using a spectrophotometer (Shimadzu brand, model IR PRESTIGE-21). The samples were prepared in solid form in potassium bromide (KBr) tablets and the data generated were treated with the aid of the origin pro 8.0 software.^29^

### 3.2. Scanning electron microscope (SEM), dispersive energy spectroscopy (EDX)

The SEM and EDX analyses were performed in a Scanning Electron Microscope, in high and low vacuum mode, Jeol, JSM-6610LV, operating at 20 kV. To perform the analysis, the samples were deposited in stubs and metallized with gold using the Denton Vacuum Desk V equipment.

### 3.3. Transmission electron microscope (TEM)

For the TEM analyses, the samples were prepared by dispersing of the TiO_2_ NPs/BMI·BF_4_ IL using isopropanol and deposited on a copper grid, coated with carbon film. The counts were performed with the aid of the Image J software.

### 3.4. X-ray diffraction (XRD)

X-ray diffraction analyses were performed using an X-ray diffractometer with a cryogenic temperatures chamber, Bruker, D8 Advance with the following parameters: voltage: 40 kV, current: 40 mA, copper tube and wavelength (*λ*): 1.5418 Å. The generated data were processed with the aid of the origin pro 8.0 software.

### 3.5. Surface area and porosity analysis (BET and BJH) analysis

The analyses to determine the specific surface area and porosity of the materials were performed through the N_2_ adsorption and desorption process with the Micromeritics Gemini VII 2390A. The volume and average size of the pores was obtained by the Barret, Joyner and Halenda method (BJH), using adsorption isotherms.

### 3.6. Difuse reflectance spectroscopy (DRS)

The optical band gap of prepared sample has been measured by using DRS measurements.^30^ These measurements have been taken in the 200–800 nm wavelength range using Scinco S-4100 UV-vis spectrophotometer.

## 4. Results and discussion

**Fig. 1:**
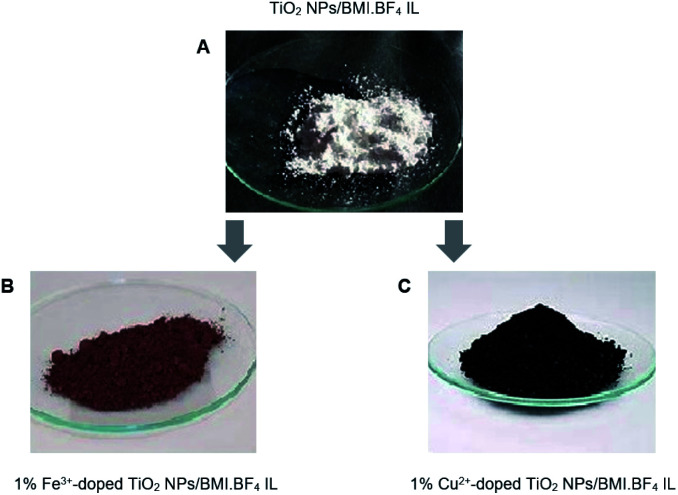
(A) TiO_2_ NPs/BMI·BF_4_ IL; (B) 1% Fe^3+^-doped TiO_2_ NPs/BMI·BF_4_ IL (C) 1% Cu^2+^-doped TiO_2_ NPs/BMI·BF_4_ IL.

The combination of non-expensive metals Cu^2+^ and Fe^3+^-doped TiO_2_ NPs in BMI·BF_4_ IL, formed new photocatalysts with different atomic ratios using the sol–gel method. The sol–gel process involves several steps, hydrolysis/condensation, gelification/polymerization, and aging and drying to form the product.^31^ The formation of the colloidal solution, sol stage, was observed through the formation of a whitish and milky solution and the gel stage by a white and viscous precipitate. The gel was subjected to drying to remove the interstitial liquid solvent, and a progressive shrinkage, tension and fragmentation of the material were observed. At the end of the drying and maceration process, a powder of different colors was obtained, which are directly related to the metal precursor/TiO_2_ NPs in BMI·BF_4_ IL atomic ratio used in the synthesis process (Fig. 1).

**Fig. 2:**
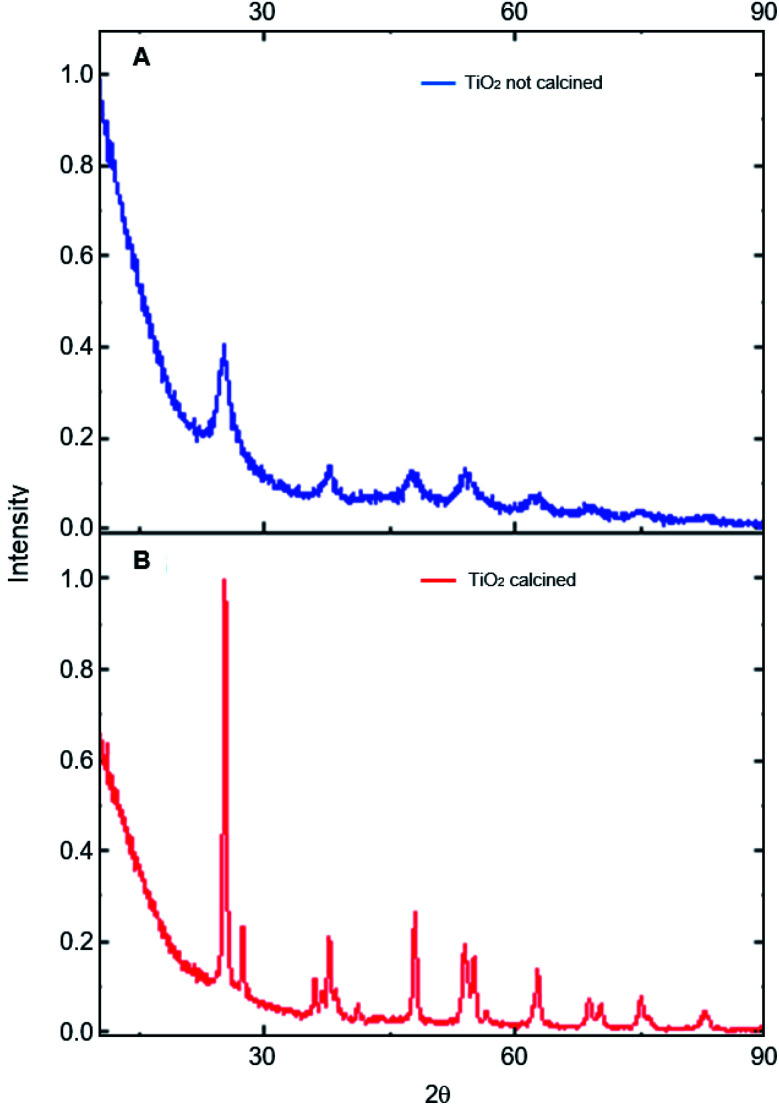
XRD analysis of: (A) TiO_2_ NPs/BMI·BF_4_ IL not calcined and (B) TiO_2_ NPs/BMI·BF_4_ IL calcined.

The photocatalysts generated were characterized by different techniques. The photocatalysts 1% Cu^2+^ and 1% Fe^3+^-doped TiO_2_ NPs/BMI·BF_4_ IL were first characterized by XRD (Fig. 2). The XRD analysis showed a composition of the anatase (55%) and rutile (45%) phases for the 1% Cu^2+^-doped TiO_2_ NPs/BMI·BF_4_ IL photocatalyst and rutile (30%), anatase (70%) phases for the 1% Fe^3+^-doped TiO_2_ NPs/BMI·BF_4_ IL photocatalyst. According to reports in the literature, the presence of phase mix tends to favor the photocatalytic activity of TiO_2_ NPS/BMI·BF_4_ IL, since it minimizes the recombination of photogenerated charges.^32^

The photocatalysts 1% Cu^2+^-doped TiO_2_ NPs/BMI·BF_4_ IL and 1% Fe^3+^-doped TiO_2_ NPs/BMI·BF_4_ IL calcined and the TiO_2_ NPs/BMI·BF_4_ IL without calcination process were characterized by XRD. The crystalline phases of the photocatalysts and the effect of calcination on the crystalline structure were analyzed. The analyses of the TiO_2_ NPs/BMI·BF_4_ IL, 1% Cu^2+^-doped TiO_2_ NPs/BMI·BF_4_ IL and 1% Fe^3+^-doped TiO_2_ NPs/BMI·BF_4_ IL photocatalysts calcined showed crystalline planes and intensities according to their crystalline phase, and being characterized by the values of the 2*θ* angles and the Miller indexes (*h k l*), characteristic of each phase of TiO_2_.

**Fig. 3:**
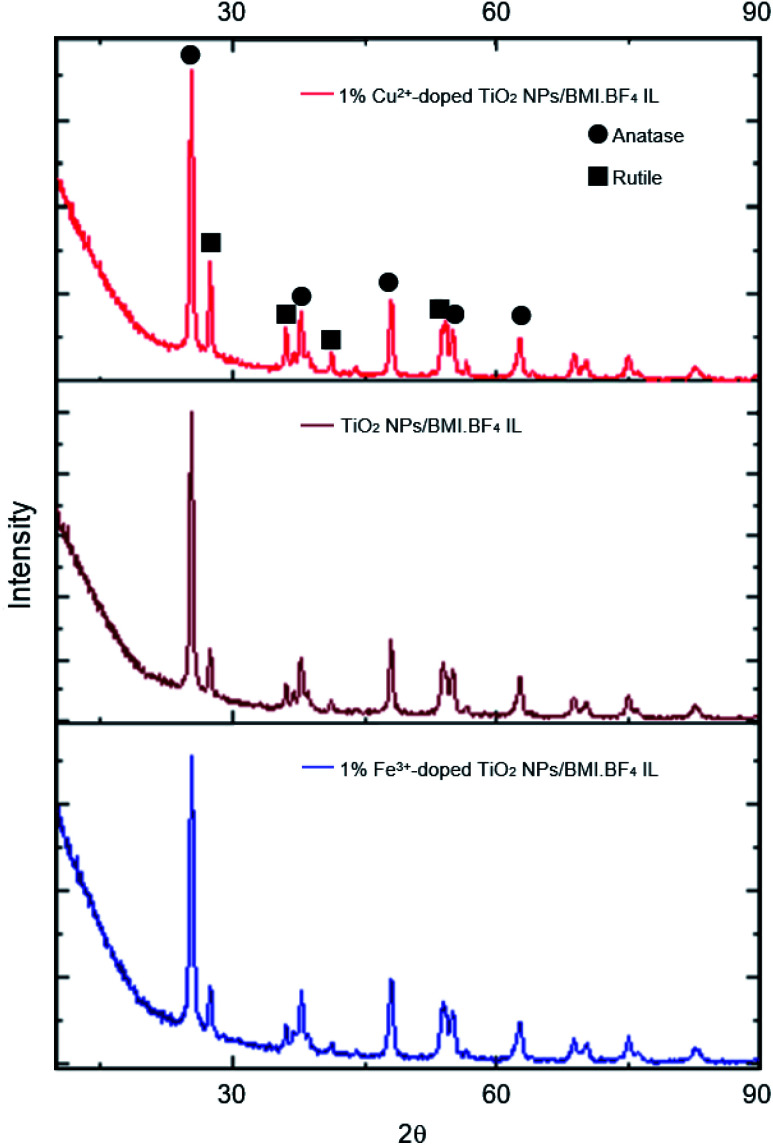
XRD analysis of: 1% Cu^2+^-doped TiO_2_ NPs/BMI·BF_4_ IL, TiO_2_ NPs/BMI·BF_4_ IL and 1% Fe^3+^-doped TiO_2_ NPs/BMI·BF_4_ IL.

In Fig. 3, it is possible to observe the planes in 25.35°, 38.16°, 48.06°, 55.03° and 62.22° corresponding to the reflections of the crystalline planes, (101), (112), (200), (211) and (204), respectively, characteristic of the anatase phase. The compounds also showed rutile crystalline phase planes at 27.68°, 36.22°, 41.65° and 54.26°, that correspond to the reflections of the crystalline planes (110), (101), (111) and (211), respectively.^33^ In all the photocatalysts analyzed, the anatase and rutile phases were observed, with the presence of the broquite crystalline phase not being observed.^34,35^ It is possible to verify that the TiO_2_ NPs without calcination do not show the characteristic planes of the crystalline phases of TiO_2_, so an amorphous material was obtained. Calcination has a fundamental role in the formation of the crystalline phases of TiO_2,_ with temperature being a determining factor in the composition of the crystalline phases of the material.

The results obtained through XRD analysis are in accordance with the results described in the literature for TiO_2_. In one study analysis of XRD reported characteristic values of the anatase phase.^34^ The values found were 25.24°, 36.98°, 48.02° and 62.74°, corresponding to the diffraction planes (101), (004), (200) and (204).^33^ In another study, diffraction planes of the anatase phase (101), (004), (103), (112), (200), (105), (211) and (204) were found, in addition to the planes of the rutile phase (110), (101) and (310).^36^ A review of XRD analysis peaks from the database of the International Committee for Powder Diffraction patterns for the crystalline anatase, rutile and broquite phase was presented. For the anatase phase, were described the patterns 25.304° (101), 33.454° (110), 38.566° (112), 48.037° (200) and 55.061° (211). For the rutile phase, the expected diffraction patterns are 27.475° (110), 36.154° (101), 41 326° (111) and 54.442° (211), and for the broquita phase, the plan 31.146° (211) was reported in the TiO_2_ NPs.

**Fig. 4:**
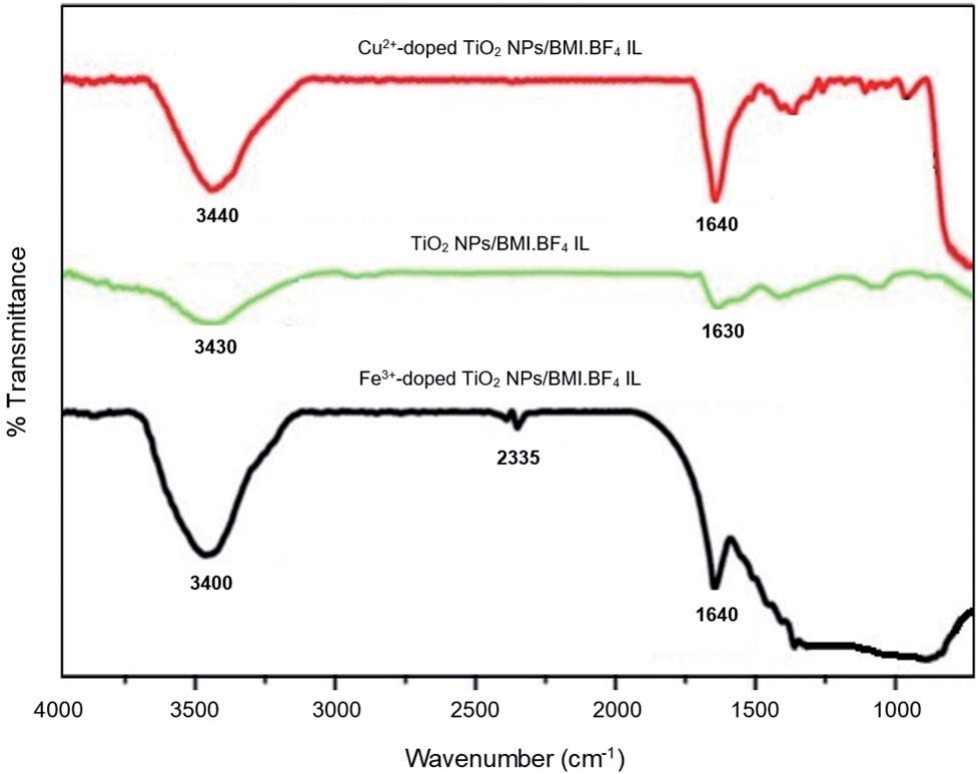
FTIR spectrum of the TiO_2_ NPs/BMI·BF_4_ IL, 1% Cu^2+^-doped TiO_2_ NPS/BMI·BF_4_ IL and 1% Fe^3+^-doped TiO_2_ NPs/BMI·BF_4_ IL.

The FTIR spectrum (Fig. 4) obtained for the photocatalysts TiO_2_ NPs/BMI·BF_4_ IL, 1% Cu^2+^-doped TiO_2_ NPs/BMI·BF_4_ IL and 1% Fe^3+^-doped TiO_2_ NPs/BMI·BF_4_ IL, exhibited a band at 1630 cm^−1^ associated with angular deformation due to adsorbed water on photocatalyst surface, HOH, *ν*_HOH_ (BMI·BF_4_ IL present is hydrophilic). The band obtained in the low energy region between 400 and 900 cm^−1^, more precisely at 750 cm^−1^, is attributed to overlaps of the vibration bands *ν*_Ti–O_ and *δ*_Ti–O–Ti_ characteristics of TiO_2_ obtained by the sol–gel method. The vibration band at 3300 cm^−1^ is related to Ti–OH, *ν*_Ti–OH_ (BMI·BF_4_ IL present is hydrophilic).^37^

For the TiO_2_ NPs/BMI·BF_4_ IL, 1% Cu^2+^-doped TiO_2_ NPs/BMI·BF_4_ IL and 1% Fe^3+^-doped TiO_2_ NPs/BMI·BF_4_ IL photocatalysts, the same infrared spectrum pattern was observed, confirming that for all cases, water adsorption on the photocatalyst surface. Another band that was observed in the FTIR spectrum is the band in the region of 2335 cm^−1^ related to Ti–O connection of TiO. This band has already been observed according to the literature.^38^

**Fig. 5:**
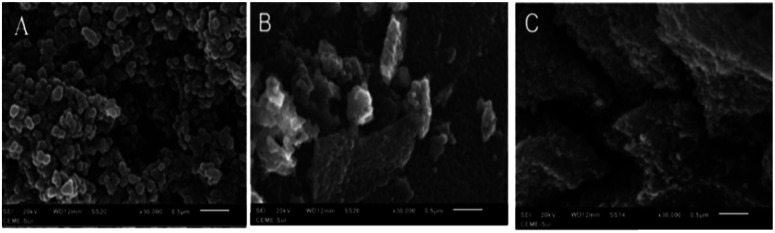
SEM micrographs obtained for samples: (A) TiO_2_ NPs/BMI·BF_4_ IL (B) 1% Cu^2+^-doped TiO_2_ NPs/BMI·BF_4_ IL and (C) 1% Fe^3+^-doped TiO_2_ NPs/BMI·BF_4_ IL.

The SEM micrographs of the TiO_2_ NPs/BMI·BF_4_ IL, 1% Cu^2+^-doped TiO_2_ NPs/BMI·BF_4_ IL and 1% Fe^3+^-doped TiO_2_ NPs/BMI·BF_4_ IL photocatalysts are exposed in Fig. 5.

**Fig. 6:**
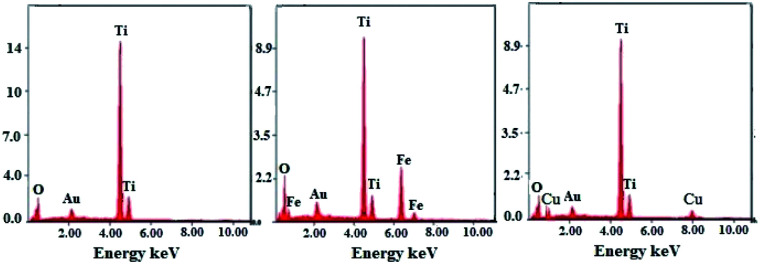
Dispersive energy spectra: (left) TiO_2_ NPs/BMI·BF_4_ IL, (middle) 1% Fe^3+^-doped TiO_2_ NPs/BMI·BF_4_ IL and (right) 1% Cu^2+^-doped TiO_2_ NPs/BMI·BF_4_ IL.

The SEM micrographs obtained for the TiO_2_ NPs/BMI·BF_4_ IL (Fig. 5A) exhibit spherical particles formation. The photocatalysts 1% Cu^2+^-doped TiO_2_ NPs/BMI·BF_4_ IL (Fig. 5B) and 1% Fe^3+^-doped TiO_2_ NPs/BMI·BF_4_ IL (Fig. 5C) showed layered and compact materials. The layered and compact surface structure presented by photocatalysts was previously described in the literature.^39,40^ The EDX analyzes were performed together with the SEM analysis for the materials TiO_2_ NPs/BMI·BF_4_ IL, 1% Cu^2+^-doped TiO_2_ NPs/BMI·BF_4_ IL and 1% Fe^3+^-doped TiO_2_ NPs/BMI·BF_4_ IL. The energy dispersion spectra obtained for the described samples can be exposed in Fig. 6.

Through the bonding energies present, it was possible to prove that only Ti and O elements were detected it the TiO_2_ NPs/BMI·BF_4_ IL photocatalyst. For the photocatalysts 1% Fe^3+^-doped TiO_2_ NPs/BMI·BF_4_ IL, only Fe, Ti, O elements were detected, and for 1% Cu^2+^-doped TiO_2_ NPs/BMI·BF_4_ IL only Cu, Ti, O elements were observed. The qualitative quantification of the chemical elements present in the described samples is shown in Table 2.

**Table 2:** Percentage mass/mass of chemical elements present in TiO_2_ NPs/BMI·BF_4_ IL, 1% Fe^3+^-doped TiO_2_ NPs/BMI·BF_4_ IL and 1% Cu^2+^-doped TiO_2_ NPs/BMI·BF_4_ IL analyzed by SEM/EDX

Material	Element chemical	%m/m
TiO_2_ NPs/BMI·BF_4_ IL	Ti	54.9
	O	36.31
1% Fe^3+^-doped TiO_2_ NPs/BMI·BF_4_ IL	Ti	46.73
	O	38.09
	Fe	0.85
1% Cu^2+^-doped TiO_2_ NPs/BMI·BF_4_ IL	Ti	50.48
	O	37.87
	Cu	0.69

**Fig. 7:**
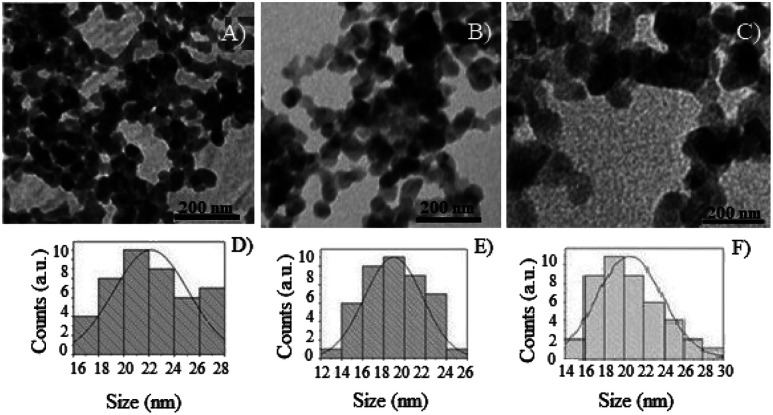
TEM analysis of the photocatalysts: (A) TiO_2_ NPs/BMI·BF_4_ IL, (B) 1% Cu^2+^-doped TiO_2_ NPs/BMI·BF_4_ IL, (C) 1% Fe^3+^-doped TiO_2_ NPs/BMI·BF_4_ IL, (D) histogram of diameter distribution TiO_2_ NPs/BMI·BF_4_ IL, (E) histogram of diameter distribution 1% Cu^2+^-doped TiO_2_ NPs/BMI·BF_4_ IL, (F) histogram of diameter distribution 1% Fe^3+^-doped TiO_2_ NPs/BMI·BF_4_ IL.

The photocatalysts were prepared using isopropanol dispersion and a drop of the obtained dispersion was deposited in a carbon-covered copper grid for TEM analysis. The micrographs obtained by TEM are exposed in Fig. 7.

Table 3 shows the average diameters obtained for TiO_2_ NPs/BMI·BF_4_ IL, 1% Fe^3+^-doped TiO_2_ NPs/BMI·BF_4_ IL and 1% Cu^2+^-doped TiO_2_ NPs/BMI·BF_4_ IL obtained by TEM analysis. Through the micrographs (Fig. 7), it is possible to verify that the TiO_2_ NPs/BMI·BF_4_ IL, as well as the 1% Fe^3+^-doped TiO_2_ NPs/BMI·BF_4_ IL and 1% Cu^2+^-doped TiO_2_ NPs/BMI·BF_4_ IL have nanometer diameter (18–22 nm), spherical shape and are preferably agglomerated. The BMI·BF_4_ ionic liquid acts as an efficient stabilizing agent for synthesis and stabilization for materials on a nanometric scale.^41–43^

**Table 3:** Average diameter and standard deviation of particles obtained for TiO_2_ NPs/BMI·BF_4_ IL, 1% Fe^3+^-doped TiO_2_ NPs/BMI·BF_4_ IL and 1% Cu^2+^-doped TiO_2_ NPs/BMI·BF_4_ IL analyzed by TEM

Material	Average particle diameter (nm)
TiO_2_ NPs/BMI·BF_4_ IL	21.8 ± 2.9
1% Fe^3+^-doped TiO_2_ NPs/BMI·BF_4_ IL	18.9 ± 2.8
1% Cu^2+^-doped TiO_2_ NPs/BMI·BF_4_ IL	20.2 ± 3.2

The average diameters of TiO_2_ NPs/BMI·BF_4_ IL obtained in this study are very similar to those reported in the literature. As an example, we can mention the synthesis of TiO_2_ NPs doped with iron that exhibited diameters in the range of 9–20 nm.^44^ In another study, the synthesis of TiO_2_ NPs formed particles with diameters in the range between 9–23 nm.^33^ In another example, the synthesis of TiO_2_ NPs doped with iron was performed at different calcination temperatures. When the calcination was at 400 °C, particles with an average diameter of 6 to 11 nm were obtained, and only the anatase phase were observed. In calcination at 600 °C a change from the anatase to rutile phase was observed, with predominance of the anatase phase, and an average diameter of 22 to 30 nm; it calcination at 800 °C, only the rutile phase was observed, with an average diameter of 50 to 100 nm.^45^

The Scherrer equation is widely applied to determine the average diameters of the TiO_2_ NPs on a manometer scale. In Table 4, based on the results, it is possible to verify that the diameters obtained from the TiO_2_ NPs/BMI·BF_4_ IL by XRD are smaller than those obtained by TEM. The difference observed between the XRD and TEM analysis indicates that the observed particles (Fig. 7) may not be a single crystalline, but clusters of isolated crystallites with a smaller particle diameter than that observed in the TEM image.^34^

**Table 4:** XRD analysis of the photocatalysts, TiO_2_ NPs/BMI·BF_4_ IL, 1% Fe^3+^-doped TiO_2_ NPs/BMI·BF_4_ IL and 1% Cu^2+^-doped TiO_2_ NPs/BMI·BF_4_ IL

Photocatalysts	XRD diameter (nm)
TiO_2_ NPs/BMI·BF_4_ IL	14.4
1% Fe^3+^-doped TiO_2_ NPs/BMI·BF_4_ IL	11.2
1% Cu^2+^-doped TiO_2_ NPs/BMI·BF_4_ IL	14.9

TEM analysis of the 1% Fe^3+^-doped TiO_2_ NPs/BMI·BF_4_ IL and 1% Cu^2+^-doped TiO_2_ NPs/BMI·BF_4_ IL also presented a smaller diameter when compared to XRD analysis. This result shows that the doping of the material with metal ions and the BMI·BF_4_ IL presence causes a decrease in the diameter of the particles. This fact occurs in relation to the incorporation of metal ions into the crystalline structure of TiO_2_ and the interaction with the organized structure of the BMI·BF_4_ ionic liquid, generating a deformation due to the different atomic sizes, such as Fe^3+^ (0.69 Ậ) and Ti^4+^ (0.745 Ậ) ions. The deformation presented in the crystal line-up results in the restriction of the growth of the Fe^3+^-TiO_2_ crystals, decreasing the diameter of the TiO_2_ crystal.^34^

Table 5 shows the percentages calculated for the crystalline anatase and rutile phases for the samples: TiO_2_ NPs/BMI·BF_4_ IL, 1% Cu^2+^-doped TiO_2_ NPs/BMI·BF_4_ IL and 1% Fe^3+^-doped TiO_2_ NPs/BMI·BF_4_ IL.

**Table 5:** Composition of the crystalline phase of photocatalysts estimated from XRD analysis

Photocatalyst	Anatase phase (%)	Rutile phase (%)
TiO_2_ NPs/BMI·BF_4_ IL	64	35
1% Cu^2+^-doped TiO_2_ NPs/BMI·BF_4_ IL	55	45
1% Fe^3+^-doped TiO_2_ NPs/BMI·BF_4_ IL	70	30

**Fig. 8:**
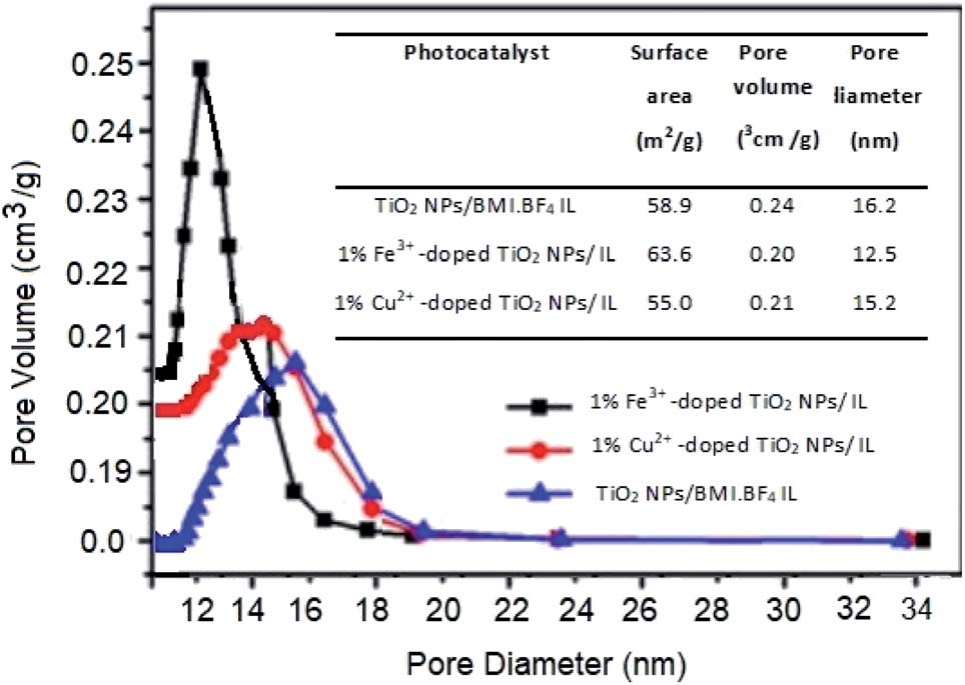
Surface area and porosity by BET and BJH analysis of the TiO_2_ NPs/BMI·BF_4_ IL_,_ 1% Fe^3+^-doped TiO_2_ NPs/BMI·BF_4_ IL and 1% Cu^2+^-doped TiO_2_ NPs/BMI·BF_4_ IL photocatalysts.

After copper doping, the crystalline phase of TiO_2_ NPs/BMI·BF_4_ IL presented a higher proportion of the rutile crystalline phase than the other materials studied. This result may be related to the use of doping with cations that present a lower number of valence electrons than the number of valence electrons of the matrix cation, thus generating anion vacancies. In other words, when TiO_2_ is doped with Cu^2+^ cations, these tend to diffuse through the structure and replace Ti^4+^ ions, moving from the O–Ti–O to Cu–O connection, thus leading to the formation of oxygen vacancies.^46,47^ The presence of oxygen vacancies favors the transition from anatase to rutile phase, because the presence of these vacancies decreases the network deformation energy that must be overcome for the rearrangement of anatase to rutile octahedrons. The unit cell of the anatase phase is composed of 24 Ti–O connections. For the formation of the rutile phase, it is necessary to break 7 bonds of the anatase phase to occur the rearrangement. With the presence of oxygen vacancies in the crystalline network of TiO_2,_ the number of Ti–O connections that need to be broken becomes smaller, thus facilitating the transition from the anatase to rutile phase in materials doped with Cu^2+^ ions. The surface area and porosity were determined by BET and BJH analysis for the TiO_2_ NPs/BMI·BF_4_ IL, 1% Fe^3+^-doped TiO_2_ NPs/BMI·BF_4_ IL and 1% Cu^2+^-doped TiO_2_ NPs/BMI·BF_4_ IL photocatalysts and the results are shown in Fig. 8.

BET analysis provided information about the surface area, the pore volume and the pore diameter values for the different photocatalysts studied. The addition of ionic liquid (IL) in TiO_2_ result in increase in surface area of the TiO_2_ nanoparticles prepared with IL.^48^

For the TiO_2_ NPs/BMI·BF_4_ IL sample, the surface area value was 58.9, for 1% Fe^3+^-doped TiO_2_ NPs/BMI·BF_4_ IL it was 63.6 and for 1% Cu^2+^-doped TiO_2_ NPs/BMI·BF_4_ IL it was 55.0.^32,49,50^ The results described that copper doping caused a minimal reduction in the surface area of the doped TiO_2_ NPs/BMI·BF_4_ IL (1% Cu^2+^ when compared to the pure TiO_2_ NPs/BMI·BF_4_ IL). The addition of metal ions to TiO_2_ NPs/BMI·BF_4_ IL did not significantly change the surface area or the pore volume of the studied materials. This fact is related to the effects of calcination temperature on the surface area and on pore diameter, which causes decreases in the surface area. The synthesized photocatalysts were calcined at constant temperature (600 °C), a fact that justifies the approximate values of surface area, volume and pore size. Based on the values obtained, the synthesized materials can be classified as mesoporous, considering that they presented pore size from 12–16 nm (Fig. 8).

Comparing the results obtained by SEM and BET analysis it was possible to observe through the SEM micrographs a different surface structure between TiO_2_ NPs/BMI·BF_4_ IL and doped photocatalysts, TiO_2_ NPs/BMI·BF_4_ IL showed spherical particles and the 1% Cu^2+^-doped TiO_2_ NPs/BMI·BF_4_ IL and 1% Fe^3+^-doped TiO_2_ NPs/BMI·BF_4_ IL exhibited a compact layered surface. However, the BET analysis exhibited that the surface area values are similar for the all photocatalysts analyzed, 58.9 m^2^ g^−1^ (TiO_2_ NPs/BMI·BF_4_ IL), 63.6 m^2^ g^−1^ (1% Fe^3+^-doped TiO_2_ NPs/BMI·BF_4_ IL) and 55 m^2^ g^−1^ (1% Cu^2+^-doped TiO_2_ NPs/BMI·BF_4_ IL). These results show that the organization of the surface structure is different, but the properties are similar.

The Cu^2+^ and Fe^3+^-doped TiO_2_ NPs/BMI·BF_4_ IL photocatalysts in different atomic metal ratios (0.5%; 1%; 5% and 10%) were first applied in phenol photodegradation to evaluate the catalytic activity. The effects of BMI·BF_4_ IL, on the photocatalytic performance of TiO_2_ showed that photogenerated electrons were the main reactive species involved in the catalytic photodegradation of phenol. The addition of BMI·BF_4_ IL enhanced the catalytic photodegradation of phenol because adsorption of [Bmim]^+^ ions on the TiO_2_ surface increased transfer of photogenerated electrons.^51^

### 4.1. Optical energy band-gap determination

**Fig. 9:**
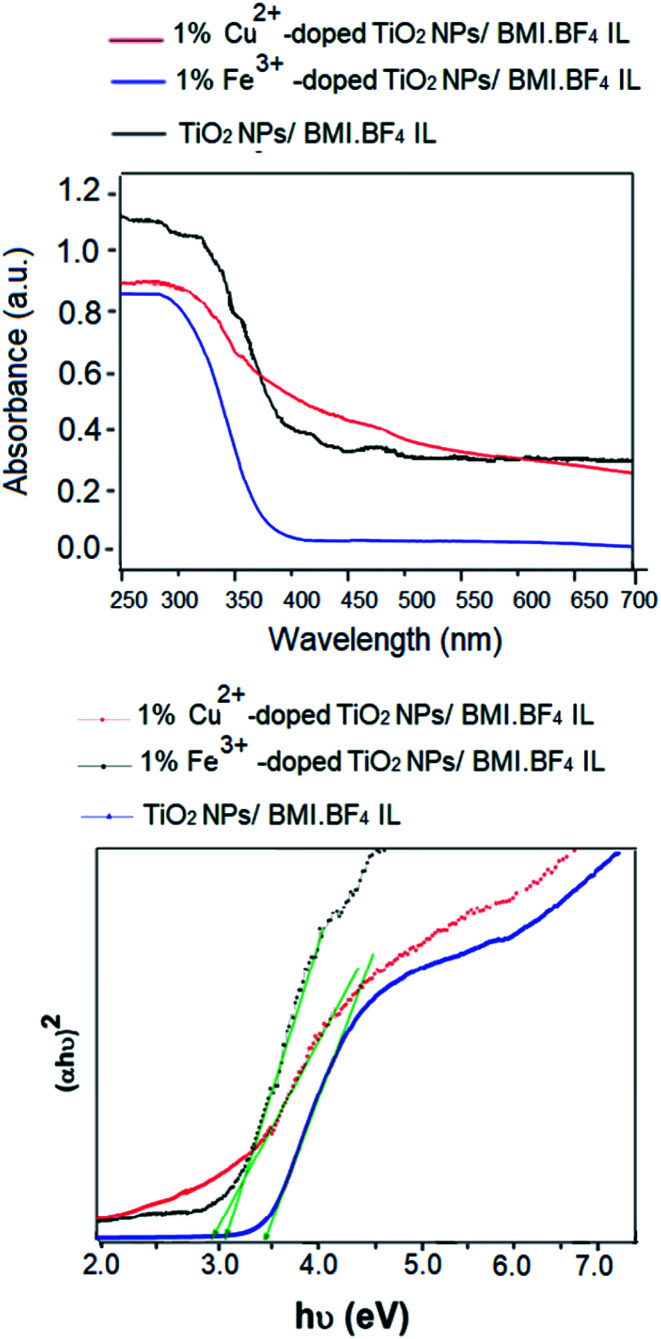
(Above) UV-vis diffuse reflectance spectra of TiO_2_ NPs/BMI·BF_4_ IL, 1% Fe^3+^-doped TiO_2_ NPs/BMI·BF_4_ IL and 1% Cu^2+^-doped TiO_2_ NPs/BMI·BF_4_ IL; (down) Tauc's plot.

The Fig. 9 show the diffuse reflectance (DR) spectra of powder of the photocatalysts (TiO_2_ NPs/BMI·BF_4_ IL, 1% Fe^3+^-doped TiO_2_ NPs/BMI·BF_4_ IL and 1% Cu^2+^-doped TiO_2_ NPs/BMI·BF_4_ IL) along with the Tauc representations. The DR spectra exhibited a large decrease in the reflectance below 400 nm that is associated to the optical absorption edge of the photocatalysts. The Tauc representations for the photocatalysts display the commonly reported linear dependence of the Kubelka–Munk function with *hv*, with a smooth function with a low curvature below and above the absorption edge. This allows an easy evaluation of the band gap as the extrapolation of the linear least squares fit of [*F*(*R*_∞_)*hv*]^1/*n*^ to zero. For materials with a well-defined absorption edge of the material, the analysis by using a double linear fitting range yields similar extrapolation values thus the main difficulty relies in the choice of the electronic transitions, as it defines the value of the *n* exponent. Indeed, the energy band gap values are quite sensitive to the selection of the fitting range and the type of electronic transitions (Fig. 9).

The band gap energy of the photocatalysts was estimated using Tauc's formula.^52^

1 (*αhν*)^2^ = *A*(*hν* − *E*_g_),

where *α* is the absorbance, and *hν* is the photon energy. The band gap energy was obtained by extrapolating the linear region of the plot (*αhν*)^2^*vs.* (*hν*) to intersect the photon energy axis (Fig. 9b). For the calculations, we have considered indirect transitions for TiO_2_ (*ca. n* = 2) and the energy band gap values of 3.1 eV, 3.3 eV, 2.8 eV was obtained for the samples of TiO_2_ NPs/BMI·BF_4_ IL, 1% Fe^3+^-doped TiO_2_ NPs/BMI·BF_4_ IL and 1% Cu^2+^-doped TiO_2_ NPs/BMI·BF_4_ IL, respectively. These results observed indicate that 1% Cu^2+^-doped TiO_2_ NPs/BMI·BF_4_ IL reduced the distance between the conduction band and valence band of TiO_2_ NPS, which could be favorable for photocatalytic reactions.^52^ The 1% Fe^3+^-doped TiO_2_ NPs/BMI·BF_4_ IL band gap energy was 3.3 eV, similar results were observed by researchers for iron.^26^

**Fig. 10:**
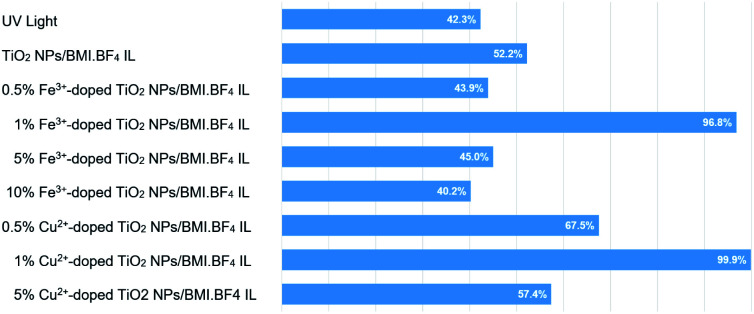
Phenol photodegradation: (a) UV light, (b)TiO_2_ NPs/BMI·BF_4_ IL, (c) 0.5% Cu^2+^, (d) 1% Cu^2+^, (e) 5% Cu^2+^, (f) 0.5% Fe^3+^, (g) 1% Fe^3+^, (h) 5% Fe^3+^ and (i) 10% Fe^3+^.

The results obtained in phenol photodegradation showed that the most active photocatalysts were 1% Cu^2+^-doped TiO_2_ NPs/BMI·BF_4_ IL (99.9%) and 1% Fe^3+^-doped TiO_2_ NPs/BMI·BF_4_ IL (96.6%) (Fig. 10). This result is due to the oxidation potential of Fe^3+^species being (−0.77 V) and Cu^2+^ species (−0.15 V). Thus, the Cu^2+^ species has a greater tendency to be oxidized by dissolved oxygen, thus initiating the photocatalysis cycle and with a greater recombination between the photogenerated electron and Cu^2+^ ions, leaving hole in the valence band more time available for the generation of OH radicals.^53^ The other photocatalysts studied; 0.5% Cu^2+^-doped TiO_2_ NPs/BMI·BF_4_ IL^,^ 5% Cu^2+^-doped TiO_2_ NPs/BMI·BF_4_ IL, 0.5% Fe^3+^-doped TiO_2_ NPs/BMI·BF_4_ IL, 5% Fe^3+^-doped TiO_2_ NPs/BMI·BF_4_ IL and 10% Fe^3+^-doped TiO_2_ NPs/BMI·BF_4_ IL did not show phenol photodegradation greater than 60%.

In catalytic processes, an important factor is the catalyst/substrate ratio. The Fig. 10 shows the influence of the catalyst concentration on phenol photodegradation. The experiments exhibited that with the increase in the concentration of the catalyst from 0.5 to 1% (m/m), the photodegradation of phenol increases (43% to 96%). This result is because the increase in the number of Fe^3+^-doped TiO_2_ NPs/BMI·BF_4_ IL or Cu^2+^-doped TiO_2_ NPs/BMI·BF_4_ IL increases the number of photons absorbed, the active sites and the number of adsorbed phenol molecules. However, there was no considerable increase in the phenol photodegradation when catalyst concentration was increased from 1% to 5% or 10%. This is attributed to catalyst agglomeration when in high concentration. The opacity and screening effect of excess Fe^3+^-doped TiO_2_ NPs/BMI·BF_4_ IL or Cu^2+^-doped TiO_2_ NPs/BMI·BF_4_ IL act as a surface layer on the surface, reducing light penetration, decreasing the active surface area, reducing photon absorption and, consequently, decreasing the catalytic activity. Therefore, the best concentration of Fe^3+^-doped TiO_2_ NPs/BMI·BF_4_ IL or Cu^2+^-doped TiO_2_ NPs/BMI·BF_4_ IL was determined as 1% (m/m).

**Fig. 11:**
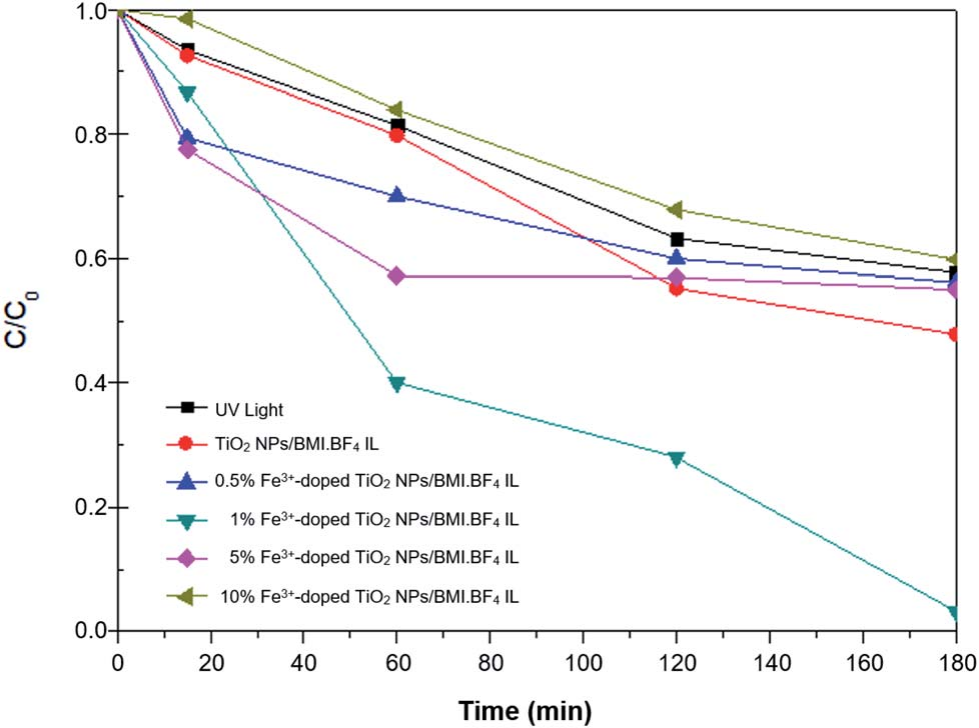
Concentration *versus* time graph (*C*/*C*_0_) of the phenol photodegradation using the photocatalysts: UV light, TiO_2_ NPs//BMI·BF_4_ IL and 0.5; 1; 5 and 10%-doped Fe^3+^ TiO_2_/BMI·BF_4_ IL.

The 1% Cu^2+^ and 1% Fe^3+^-doped TiO_2_ NPs/BMI·BF_4_ IL synthesized using the sol–gel method at 90 °C and calcined at 600 °C during 1 h generated the photocatalytic materials. The phenol photodegradation as a function of time (0–180 min) was investigated by applying the following photocatalysts; UV light, TiO_2_ NPs/BMI·BF_4_ IL, 1%, 5% and 10% Fe^3+^-doped TiO_2_ NPs/BMI·BF_4_ IL. The displayed curves clearly exhibit the higher catalytic efficiency obtained by the 1% Fe^3+^-doped TiO_2_ NPs/BMI·BF_4_ IL photocatalyst, compared to the other given photocatalysts (Fig. 11).

The results of phenol photodegradation showed a degradation of around 42.3% when only UV light was used. In comparison, the use of photocatalyst TiO_2_ NPs/BMI·BF_4_ IL exhibited higher photocatalytic activity (52.2%) than that obtained using only UV light. This fact is related to TiO_2_ NPs/BMI·BF_4_ IL present valence bands (VB) and conduction bands (CB) separated by a well defined energy. In the presence of visible light, the photocatalyst has its VB electrons excited and promoted to CB, generating the electron/hole pair (h^+^). The hole formed is extremely oxidative, presenting a potential to generate HO˙ radicals (which is even greater due to the presence of BMI·BF_4_ IL), from water molecules adsorbed on the surface of TiO_2_, thus degrading phenol to CO_2_ and H_2_O.^54^

The application of the 1% Fe^3+^-doped TiO_2_ NPs/BMI·BF_4_ IL photocatalyst exhibited high phenol degradation rates (96.8%), with the high degradation potential of the 1% Fe^3+^-doped TiO_2_ NPs/BMI·BF_4_ IL photocatalyst due to rapid regeneration of the electron–hole pair. TiO_2_ NPs/BMI·BF_4_ IL doped with iron ions forms a cycle where dissolved oxygen oxidizes iron, forming the O_2_˙^−^ species, which with the help of H^+^ present in the solution, form the HO˙ radical. In this process, the photogenerated electrons reduce the iron, not quickly returning to the BV of the photocatalysts and leaving the hole generated in the BC available for a longer time.^54^ The 5% Fe^3+^-doped TiO_2_ NPs and 10% Fe^3+^-doped TiO_2_ NPs photocatalysts exhibited low photocatalytic activity, with values similar to those obtained with the percentage of degradation when only UV light was applied (40–45%). This fact indicates that doping with 5% and 10% of iron made the photocallyzer inactivated. According to literature data, the solubility of iron in TiO_2_ is 1% m/m, thus the higher concentration of iron ions makes diffusion in the TiO_2_ structure impossible (even with the presence of BMI·BF_4_ IL, which improves porosity of TiO_2_), leading to agglomeration on the TiO_2_ surface and formation of Fe_2_O_3_. The formation of agglomerates of Fe_2_O_3_ cause a reduction of the active catalytic centers decreasing the photocatalytic activity.^55^

The use of 0.5% Fe^3+^-doped TiO_2_ NPs/BMI·BF_4_ IL photocatalysts showed an increase in photocatalytic activity initially, but after this period (15 min) the photocatalyst activity was statistically equivalent (*t* test, 95% confidence) to TiO_2_ NPs/BMI·BF_4_ IL photocatalyst activity (45%).^27^ In the literature, results similar to those obtained in this work were observed. When comparing the different concentrations of TiO_2_ NPs doped with iron ions, the material with 1% Fe^3+^ showed the best photocatalytic activity. The effect of 1% Cu^2+^-doped TiO_2_ NPs/BMI·BF_4_ IL photocatalyst was also investigated in this work. Table 6 shows the degradation percentages obtained for phenol.

**Table 6:** Effect of photocatalysts (UV light; TiO_2_ IL; 0.5%, 1%, 5%, and 10% Fe^3+^/IL; and 1% Cu^2+^/IL) on the percentage of phenol degradation as a function of time

Time (min)	% Phenol photodegradation						
	UV light	TiO_2_ IL	0.5% Fe^3+^	5% Fe^3+^	10% Fe^3+^	1% Fe^3+^	1% Cu^2+^
0	100	100	100	100	100	100	100
15	93.5	92.6	79.4	77.5	98.5	70.0	87.5
60	81.4	79.8	70.0	57.2	83.9	45.4	78.7
120	63.2	55.2	58.9	56.9	67.8	38.0	10.3
180	57.7	47.8	56.1	55.0	59.8	32.5	0.1

**Fig. 12:**
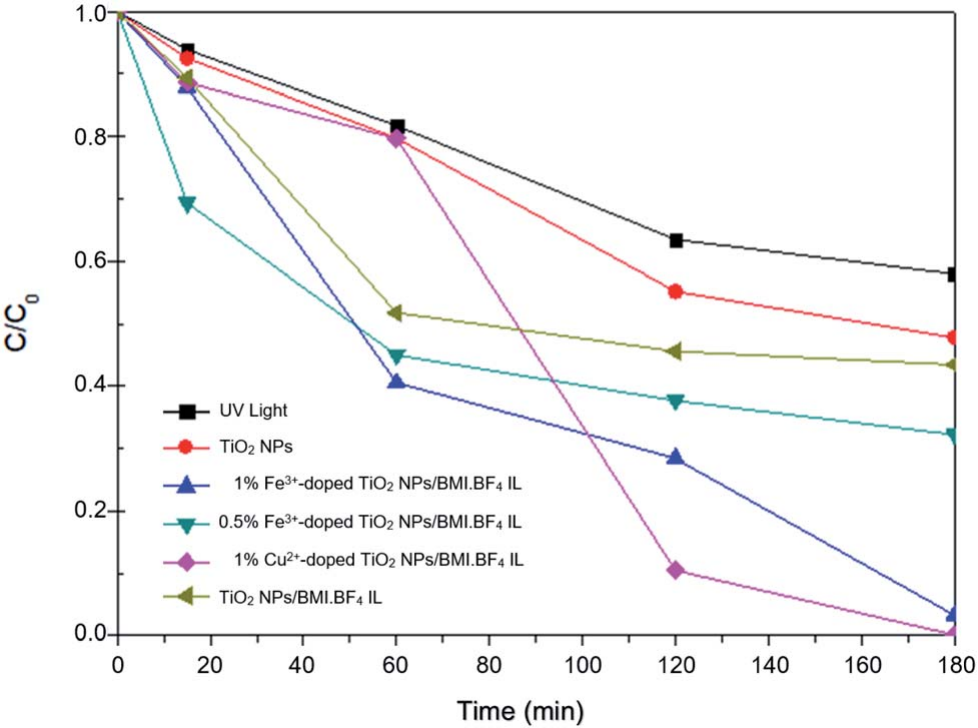
Graph of *C*/*C*_0_ concentration as a function of time for phenol photodegradation by different catalysts.

The curves shown in Fig. 12 exhibit the results of phenol degradation using the photocatalysts: UV light, TiO_2_ NPs/BMI·BF_4_ IL, 1% Fe^3+^-doped TiO_2_ NPs/BMI·BF_4_ IL, and 1% Cu^2+^-doped TiO_2_ NPs/BMI·BF_4_ IL.

### 4.2. Kinetic studies of phenol photodegradation

**Fig. 13:**
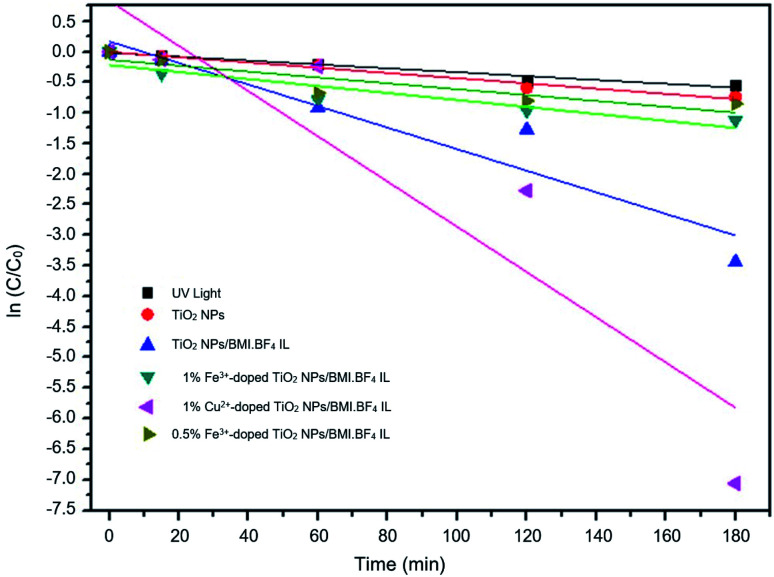
Graph of investigated photocatalysts in phenol photodegradation expressed by a first-order kinetic equation.

**Fig. 14:**
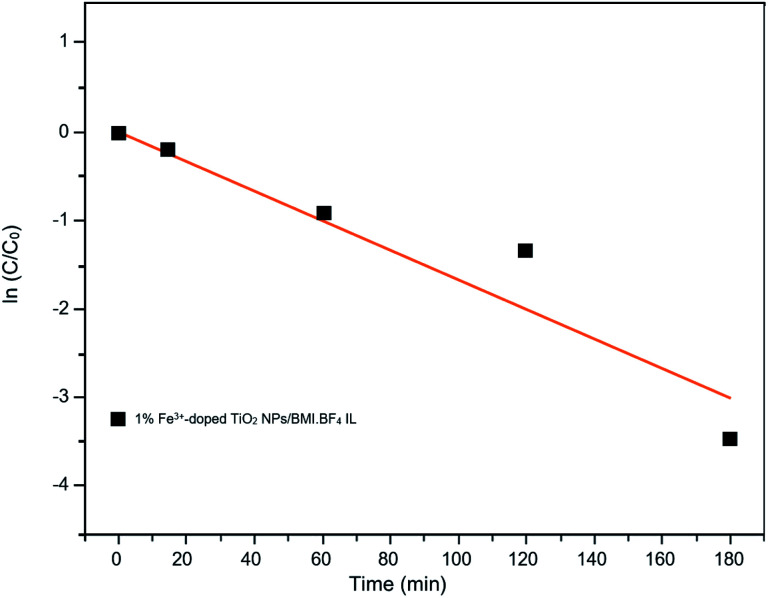
Graph of the great catalyst 1% Fe^3+^/TiO_2_ NPs/BMI·BF_4_ IL in phenol photodegradation expressed by a first-order kinetic equation (*R*^2^ = 0.91).

**Fig. 15:**
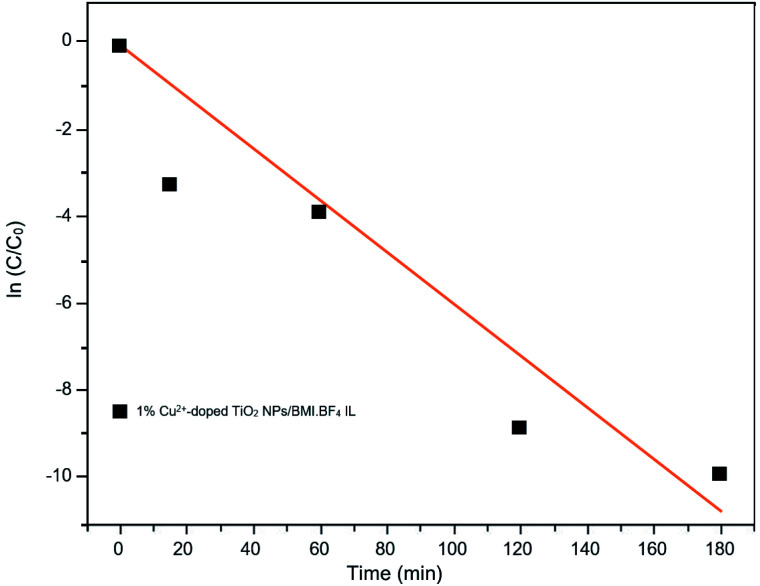
Graph of the great catalyst 1% Cu^2+^-doped TiO_2_ NPs/BMI·BF_4_ IL in phenol photodegradation expressed by a zero-order kinetic equation (*R*^2^ = 0.93).

Kinetic studies of the investigated photocatalysts were also carried out (Fig. 13–15). From the results obtained in the kinetic studies of phenol photodegradation, it was possible to observe the obtainment of first-order kinetics for the photocatalysts: UV light, TiO_2_, 1% Fe^3+^-doped TiO_2_ NPs/BMI·BF_4_ IL and TiO_2_ NPs/BMI. BF_4_ IL. Fig. 13 shows the graphs obtained for the first-order kinetic equations for the phenol compound. The 1% Fe^3+^-doped TiO_2_ NPs/BMI·BF_4_ IL photocatalyst showed a first-order rate law was observed (Fig. 14) and the 1% Cu^2+^-doped TiO_2_ NPs/BMI·BF_4_ IL photocatalyst, exhibited a zero-order rate law was observed (Fig. 15).

As shown in Fig. 15, it is possible to verify that for the 1% Cu^2+^-doped TiO_2_ NPs/BMI·BF_4_ IL catalyst, the photodegradation followed a zero-order velocity law. The kinetic degradation curves obtained for the phenol compound using catalysts were those in which the *R*^2^ correlation coefficient was closer to one (1), according to eqn (2).^56^

This parameter was chosen to evaluate the degradation kinetic curves, because the correlation coefficient measures the fraction of the variation observed in *y*, that is, the closer the points are to the line predicted by least squares analysis, the smaller are the residues observed for the experimental model.^57^2
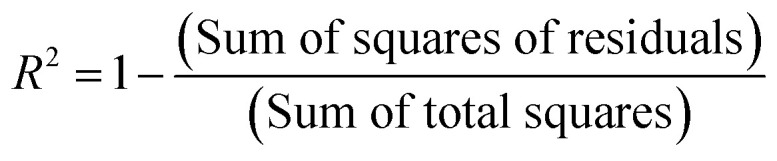


Table 7 shows the correlation coefficients for phenol with zero-order and first-order reaction kinetics for the photocatalysts: UV light, TiO_2_ NPs/BMI·BF_4_ IL, 1% Fe^3+^-doped TIO_2_ NPs/BMI·BF_4_ IL, 1% Cu^2+^-doped TIO_2_ NPs/BMI·BF_4_ IL.

**Table 7:** Correlation coefficient for the kinetic equations of phenol photodegradation

Photocatalyst	Phenol photodegradation	
	Zero-order	First-order
UV light	0.96	0.97
TiO_2_ NPs/BMI·BF_4_ IL	0.97	0.98
1% Fe^3+^-doped TiO_2_ NPs/BMI·BF_4_ IL	0.77	0.91
1% Cu^2+^-doped TiO_2_ NPs/BMI·BF_4_ IL	0.93	0.85

Through the data shown in Table 7, it is possible to observe that the best correlation coefficients for phenol were with the 1% Cu^2+^-doped TiO_2_ NPs/BMI·BF_4_ IL photocatalyst, which exhibited a zero-order equation and an *R*^2^ of 0.93, values greater than those obtained by the first-order equation, which showed *R*^2^ values around 0.85. The other photocatalysts applied to phenol presented *R*^2^ close to one (1) for the first order equation. Literature data reported the photocatalytic decomposition of phenol following pseudo zero-order reaction kinetics for TiO_2_ doped with copper ions. In addition, Khraisheh also found that TiO_2_ doped with copper ions has a higher percentage of degradation when compared to TiO_2_ doped with Fe and Mn 1% m/m.^58^ In another study, it was described that degradation of phenol followed the first order kinetics with the use of TiO_2_ photocatalyst doped with Fe^3+^ ions.^59^ In this same work, it was highlighted that the doping of TiO_2_ with Fe^3+^ ions promotes an increase in the photocatalytic activity in the degradation of phenol when compared to pure TiO_2_, and that this result is due to the recombination of Fe^3+^ charges and the TiO_2_ e^−^/h^+^ pair.^59^

### 4.3. Phenol photodegradation studies in wastewater from the tobacco industry

The phenol photodegradation was also carried out in an wastewater from the tobacco industry, the wastewater contains, in addition to phenol, other compounds such as alkaloids, specific nitrosamines (TSNAs), metals, metalloids, acetones, pesticide residues, humectants, benzo[*a*]pyrene (B[*a*]P), radionuclides and alphatoxins, benzene, naphthalene, pyridine, among other compounds.^60^

The studied industrial wastewater was exposed to the photocatalysis process without any pre-treatment. The two photocatalysts that showed the best photocatalytic activity in the standard phenol sample, 1% Cu^2+^-doped TiO_2_ NPs/BMI·BF_4_ IL and 1% Fe^3+^-doped TiO_2_ NPs/BMI·BF_4_ IL, were investigated in phenol degradation in industrial wastewater. The results obtained are shown in Table 8. Through the exposed data, it is observed that the photocatalyst 1% Fe^3+^-doped TiO_2_ NPs/BMI·BF_4_ IL exhibited the best photocatalytic activity, with degradation values of 60.2% for the phenol compound in tobacco industry wastewater and the 1% Cu^2+^-doped TiO_2_ NPs/BMI·BF_4_ IL exhibited slightly lower phenol photodegradation with value of 56.7%.

**Table 8:** Phenol degradation in tobacco industry wastewater

Time	Photocatalyst	
	1% Fe^3+^-doped TiO_2_ NPs/BMI·BF_4_ IL	1% Cu^2+^-doped TiO_2_ NPs/BMI·BF_4_ IL
0	100	100
15	88.2	93.0
60	67.0	79.7
120	64.2	71.2
150	62.2	63.8
180	60.2	56.7

The lower values of phenol degradation present in industrial effluent, when compared to values obtained for standard samples, can be related to the high complexity of the tobacco matrix, which is composed of more than 4700 organic and inorganic compounds.^60^ The complexity of the tobacco matrix and the fact that an effluent without pretreatment was used may have generated competition by the HO˙ radicals present in the medium, which probably acted as oxidizing agents for other organic species existing in the matrix.^61^

## 5. Conclusions

The synthesis of the photocatalysts under UV/vis irradiation by the sol–gel method, using non-expensive metals Fe^3+^ and Cu^2+^-doped TiO_2_ NPs/BMI·BF_4_ IL, proved to be simple and effective in generating highly active catalysts in phenol photodegradation (99.9%).

The active TiO_2_ NPs, 1% Cu^2+^ and 1% Fe^3+^-doped TiO_2_/BMI·BF_4_ IL NPs photocatalysts were been characterized by different techniques. The FTIR analysis showed the presence of characteristic bands for the formation of the connections *ν*_Ti_–_O_ and *δ*_Ti–O–Ti_ in the analyzed photocatalysts, confirming the formation of the TiO_2_ NPs. BET and BJH analysis showed a similar surface area (55–63 m^2^ g^−1^) and pore diameter 12–16 nm. XRD analysis confirmed the presence of two crystalline phases of TiO_2_, anatase (majority) and rutile, with particle diameter of 11–14 nm. SEM micrographs showed spherical particles formation for TiO_2_ NPs and compact layers for 1% Cu^2+^ and 1% Fe^3+^-doped TiO_2_ NPs. EDX analysis confirmed only the presence of Ti, O, Fe and Cu in the analyzed photocatalysts. The TEM images exhibited spherical shape, for all photocatalysts with an average diameter of 18–22 nm. The smaller particle diameter observed by XRD occurs in relation to the incorporation of metal ions into the crystalline structure of TiO_2_, causing a decrease in the diameter. DRS analysis and Tauc equation exhibited energy band gap values of 3.1 eV, 3.32 eV, 2.78 eV by TiO_2_ NPs/BMI·BF_4_ IL, 1% Fe^3+^-doped TiO_2_ NPs/BMI·BF_4_ IL and 1% Cu^2+^-doped TiO_2_ NPs/BMI·BF_4_ IL, respectively. The various concentration of photocatalysts were tested, but 1% Cu^2+^-doped TiO_2_ NPs/BMI·BF_4_ IL and 1% Fe^3+^-doped TiO_2_ NPs/BMI·BF_4_ IL exhibited high catalytic activity (99.9% and 96.8, respectively) in phenol photodegradation. The best photocatalysts 1% Cu^2+^ and 1% Fe^3+^-doped TiO_2_ NPs/BMI. BF_4_ IL were also investigated in an industrial wastewater from a tobacco industry, the results showed 56.7% and 60.2% respectively, of phenol degradation due to the complexity of the tabacco matrix wastewater.

## Author's contribution

Daiane Kessler Fischer (Master's student): synthesis, characterization and application of photocatalysts. Karina Rodrigues de Fraga (Graduation student): characterization and application of photocatalysts. Carla Weber Scheeren (PhD): guidance in the development of work and writing and work organization.

## Conflicts of interest

There are no conflicts to declare.

## Supplementary Material
